# Scale-down optimization of a robust, parallelizable human induced pluripotent stem cell bioprocess for high-throughput research

**DOI:** 10.1016/j.btre.2025.e00900

**Published:** 2025-05-22

**Authors:** James Colter, Tiffany Dang, Julia Malinovska, Jessica May Corpuz, Dora Modrcin, Roman Krawetz, Kartikeya Murari, Michael Scott Kallos

**Affiliations:** a Pharmaceutical Production Research Facility (PPRF), University of Calgary, 2500 University Drive NW, Calgary, AB T2N 1N4, Canada; b Integrated Circuits and Optical Imaging Laboratory (ICOI), University of Calgary, 2500 University Drive NW, Calgary, AB T2N 1N4, Canada; c Department of Biomedical Engineering, Schulich School of Engineering, University of Calgary, 2500 University Drive NW, Calgary, AB T2N 1N4, Canada; d McCaig Institute for Bone and Joint Health, University of Calgary3280 Hospital Drive NW, Calgary, AB T2N 4Z6, Canada; e Department of Biochemistry and Molecular Biology, University of Calgary, 2500 University Drive NW, Calgary, AB T2N 1N4, Canada; f Alberta Children’s Hospital Research Institute, University of Calgary, 3330 Hospital Dr. NW, Calgary, AB T2N 4N1, Canada

**Keywords:** Human induced pluripotent stem cells, Stem cell bioprocessing, Optimization, Stirred Tank Bioreactors

## Abstract

Human induced pluripotent stem cell (hiPSC) derived therapeutics require clinically relevant quantities of high-quality cell populations for applications in regenerative medicine. The lack of efficacy exhibited across clinical trials suggests deeper understanding of the networks governing phenotype is needed. Further, costs limit study throughput in characterizing the artificial niche relative to outcomes. We present herein an optimized strategy to enable high-throughput hiPSC expansion at <20 mL research scale. We assessed viability of single cell inoculation and aggregate preformation to facilitate proliferation. We modeled aggregate characteristics against agitation rate. Our results demonstrate tunable control with fold expansion comparable to commercial systems. Marker quantification and teratoma assay confirm functional pluripotency. This approach constitutes a scalable protocol to accelerate hiPSC research, and a significant step in advancing the rate of progress in elucidating links to derivative functionality. This work will enable statistically rigorous studies targeting hiPSC and downstream phenotype for clinical manufacturing.

## Highlights

• Preformation of aggregates tuned by cell density enable cultivation of hiPSCs in scale-down shear environments.
• Scale-down systems utilizing preformation protocols achieve comparable fold expansion with commercial systems.
• Expression of pluripotency markers and functional differentiation capacity is maintained following passage in scale-down culture.
• Successful application of hiPSC protocols at < 20 mL scales enable rapid and cost-effective research into cell phenotype under dynamic conditions.

## Graphical abstract

Implementation of adapted protocols enable scale-down systems as a tool for high-throughput iPSC biomanufacturing research, in platforms conducive to scale-up for clinical manufacturing.

**Figure:**
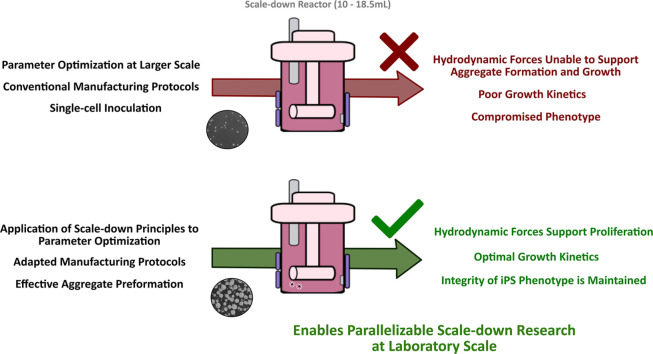

## 1 Introduction

The global market for human induced pluripotent stem cell (hiPSC) derived products has skyrocketed in the last decade [1]. Human iPSCs are used as cell source starting material for organoid development, as tools to study disease and development in research, and in approaches to cell and gene therapy – providing a legal and ethical alternative to the use of embryonic stem cells across the globe [[2], [3], [4]]. However, in focusing efforts to progress cell therapies as global solutions in clinical medicine, a common theme presents in clinical trial outcomes – limited effectiveness of downstream cell therapeutics in treating disease or dysfunction [5]. This is one of the most difficult challenges to overcome in envisioning the evolution of clinical approaches to integrate cell therapy as a global standard for healthcare.

Human iPSCs have distinct characteristics from their naturally occurring embryonic counterparts [[6], [7], [8]]. Somatic memory plays an integral role in forming the epigenetic basis for these differences, suggesting a critical role in the induction strategy and networks targeted in recapitulating embryonic state [9]. This is coupled to donor characteristics and exacerbated by the environment in which these cells are maintained during and following induction [10,11]. Conventional manufacturing strategies encompass multi-passage clonal expansion to generate cell numbers relevant for clinical use [12]. It is important to note that this phenotypic stasis is not exhibited in nature, as pluripotency exists along a dynamic phenotypic trajectory throughout development of the blastocyst, where pluripotent phenotype is present only for a few days [13]. The maintenance and spatiotemporal orchestration of differentiation of pluripotent phenotype is strictly governed by the embryonic niche. While the conventional approach to adapting artificial niches for expansion of PSCs has primarily focused on scaling cell density while maintaining core pluripotent identity, adaptation of the cells to their environment, re-organization of niche response, and gene-regulatory network reinforcement through factors undoubtedly have broader phenotypic implications downstream [10,[14], [15], [16]].

A present obstacle to the implementation of advanced optimization strategies for expansion bioprocessing is the adaptability of protocols developed in research, and the success of translation across process infrastructures and downstream targets. Our group is most interested in better understanding how networks that govern phenotype are influenced by environmental factors, parametric setpoints, and control strategies that ultimately help shape phenotypic phenomena in hiPSC populations throughout the expansion process. Given limitations of larger scale systems in generating cost-effective quantities of biological replicates for research and facilitating integration with rigorous population-relevant phenotypic characterization (i.e. donor variability, batch variability, induction source material, induction strategy, medium compositions, etc.), we endeavored to optimize a system at research scale for the purpose of better understanding and modeling phenotypic interactions. Previous work by our group and others has enabled efficient scale-up using computational fluid dynamics and empirical study to inform relevant reactor parameters for translation from laboratory scale to clinical manufacturing, providing tools to bridge the gap between our findings in a scale-down environment and large-scale clinical operations [[14], [15], [16], [17], [18], [19]]. This line of reasoning provides the foundation for our approach – to facilitate the acquisition of biologically relevant information at a scale that allows for rapid discovery, mindful of the engineering constraints of manufacturing scale-up.

In this work, our team has successfully optimized a scale-down system (10–18.5 mL) enabling high parallelizability and low-cost bioprocessing of hiPSCs that follows principles conducive to scale-up. The purpose of this research is to present findings concerning successful scale-down protocol optimization that facilitate ease of experimentation for high-quality hiPSC expansion bioprocesses at a small fraction of the costs incurred at larger scales. Our hope is that these findings enable our group and like-minded researchers to explore cell state dynamics stemming from environmental setpoints and process perturbations to elucidate their implications for downstream therapeutic derivation, and ultimately contribute to the realization of cell therapy as a revolutionary approach to clinical medicine.

## 2 Materials and methods

Scale-down optimization was carried out using protocols adapted from our previous work optimizing hiPSC bioprocessing at 100 mL scale [15,19]. Adaptations to existing methodologies are reported herein. A 10 µM concentration of Y27632 rho kinase inhibitor (#72,302, StemCell Technologies) was used for all noted supplementations. All procedures were performed in compliance with relevant laws and institutional guidelines and were approved by the Conjoint Health Research Ethics Board (CHREB) at the University of Calgary under ethics protocol #REB14–1914. All animal handling and experimental procedures were performed in accordance with the Canadian Council of Animal Care guidelines and approved by the University of Calgary Animal Care Committee (protocol AC24–0033).

### 2.1 Cell source starting material

Human induced pluripotent stem cell (hiPSC) line PGPC14 XX-chromosome was kindly provided by the Ellis lab at the University of Toronto at passage 19 [20,21]. Cells were propagated to passage 21 in static adherent vessels and cryopreserved prior to this study at a density of 1 × 10^6^ cells/vial in 1 mL mFreSR (#05,855, StemCell Technologies).

### 2.2 Cell thaw and resuspension for static inoculation

Vials containing cryopreserved hiPSCs were removed from liquid nitrogen and left at ambient room temperature for 5 min to reduce the temperature gradient observed in transitioning from cryopreservation to water bath submersion [22]. Vials were then placed in a 37 °C water bath until ice crystals were barely visible. Cells in 1 mL thawed suspension were then resuspended droplet by droplet in a conical tube containing 4 mL mTeSR1 + Y27632 by serological pipette. This suspension was centrifuged at 300 × *g* for 5 min. Supernatant was discarded, and cells were resuspended in 5 mL mTeSR1 + Y27632 by serological pipette prior to counting and inoculation.

### 2.3 Static culture

Cells were cultured in static T-25 culture flasks (#156,367, Thermo Fisher) for 4 days in advance of aggregate preformation. A 3 mL aliquot of substrate solution containing Vitronectin-XF (#07,180, StemCell Technologies) at a concentration of 10 µg/mL in CellAdhere dilution buffer (07,183#, StemCell Technologies) was applied per flask prior to inoculation. Flasks were kept in sterile conditions at room temperature for 1 hour to facilitate surface binding. Flasks were then sealed with parafilm and refrigerated (2–8 °C) for 24 h. Following refrigeration, parafilm was removed and remaining solution aspirated. Culture medium was inoculated, and flasks were incubated at 37 °C for 45 min. Cells were thawed and inoculated on Day −4 at a concentration of 2 × 10^4^ cells/cm^2^ in 5 mL mTeSR1 (85,850, StemCell Technologies) + Y27632. A 50 % medium replacement with mTeSR1 was performed daily to dilute inhibitor concentration and maintain optimal growth conditions. Cell inoculation density and feeding regime were implemented to facilitate healthy colony formation not exceeding 600 µm in diameter prior to harvest.

### 2.4 Static harvest

Cells were harvested from static by first aspirating the culture medium, rinsing with 1X PBS, and inoculating with 3 mL Accutase + Y27632 (#07,922, StemCell Technologies) per flask. Inoculated flasks were left to incubate for 10 min. Following incubation, cells in Accutase were diluted 50 % in mTeSR1 + Y27632 and transferred to a conical tube for centrifugation at 300 × *g* for 5 min. Cells were resuspended in 10 mL mTeSR1 + Y27632 following aspiration of supernatant by serological pipette. Sample aliquots of 200 µL were taken by single-channel pipette for counting prior to aggregate preformation.

### 2.5 Aggregate preformation

Following static harvest, aggregates were preformed by inoculating cells in non-adherent 6-well plates (Cat#657–185, Greiner CELLSTAR) in 2 mL/well mTeSR1 + Y27632, and incubating plates for up to 24 h at cell densities of 1 × 10^4^ to 4 × 10^4^ cells/cm^2^. The cell suspension was gently triturated 20x to facilitate uniform cell distribution prior to allowing the cells to self-aggregate. Aggregates were imaged following preformation, prior to inoculation in stirred suspension.

### 2.6 Dynamic culture in scale-down system

Aggregates preformed at a density of 1 × 10^4^ cells/cm^2^ were transferred into stirred suspension reactors by 2 mL serological pipette. Agitation was controlled at one of three speeds: 100 RPM, 120 RPM, or 150 RPM using a bioWiggler programmable stirrer platform (VWR, USA). An inoculation density of 3.6 × 10^4^ cells/mL was used for all agitation rates. Cultures were carried out over 3 days following inoculation (for a total of 4 days including aggregate preformation). A total culture time of four days was chosen due to the batch experimental design for assessing culture optimization. Static controls were maintained for all experiments under the protocols described above. Vessel components and scale of dimension are shown in supplementary Figure 3.

### 2.7 Aggregate harvest and dissociation

Prior to harvesting aggregates, 2 mL samples were collected during controlled agitation for imaging. Following sampling, the entire culture medium was aspirated to a conical tube and centrifuged at 300 × *g* for 5 min. Supernatant was aspirated, and 1 mL of Accutase added. Conical tubes were incubated in a 37 °C water-bath for 10 min, after which 1 mL of mTeSR1 + Y27632 was added to neutralize enzymatic activity. The solution was triturated 20x to facilitate a homogenous single-cell suspension, and centrifugation was repeated. Supernatant was then removed, and cells were resuspended in 10 mL mTeSR1 + Y27632 and triturated into single-cell suspension for counts and further characterization.

### 2.8 Imaging and quantification

All imaging was performed using a Zeiss AxioScope.A1 under phase contrast and processed in AxioVision software (Zeiss, Germany). Static images were taken of the adherent flask surface prior to harvest, and aggregate images were taken in 12-well plates following pre-harvest sampling. Cell counts were taken by automated fluorescence-based nucleus counts using a NucleoCounter and processed in NucleoView (ChemoMetec, Denmark). Three 100 µL samples were quantified to obtain a mean population count. Aggregate distributions were estimated in Python with OpenCV by applying a median blur to phase-contrast images and using a Hough gradient algorithm to estimate aggregate radii within each image. A total of 6 images taken on day 4 for each biological replicate were used to model distributions for the agitation rates explored.

### 2.9 Flow cytometry

Quantification of marker expression was carried out using a Cytek Aurora System (Cytek Biosciences, USA) and analyzed in the acquisition software or FlowJo (BD Lifesciences, USA). A panel of six markers (surface and nuclear) were selected to profile phenotype by assessing OCT4 (NB100–2379AF405, Bio-Techne), Sox2 (IC20181N, Bio-Techne), Nanog (MA1–017-D488, Thermo Fisher), SSEA4 (46–8843–42, Thermo Fisher), TRA-1–60 (12–8863–82, Thermo Fisher), and TRA-1–81 (17–8883–42, Thermo Fisher). Samples containing ∼1 × 10^6^ cells were centrifuged at 500 × *g* for 5 min. Following aspiration of supernatant with a single-channel pipette, cells were resuspended in 200 µL of a 1:4 dilution of IC fixation buffer (00–8222–49, Thermo Fisher) in 1x phosphate buffered saline (PBS) (10,010,023, Thermo Fisher). Cells in fixative were refrigerated (4 °C) for 20 min in the dark. Cells in solution were then diluted 1:2 in PBS, and centrifuged at 500 × *g* for 5 min. Following supernatant aspiration, cells were resuspended in a 1:10 dilution of IC permeabilization buffer (00–8333–56, Thermo Fisher) and refrigerated (4 °C) for 20 min in the dark. The permeabilized cell solution was then diluted 1:5 in PBS and centrifuged at 500 × *g* for 5 min. Cells were then resuspended in 100 µL of 3 % w/v bovine serum albumin (BSA) (A9647, Thermo Fisher) in PBS, containing 1 µL per sample of each fluorophore-conjugated antibody. Antibodies were excluded from the staining buffer for unstained controls. Samples in staining buffer were refrigerated (4 °C) for 1 h in the dark. Samples were then diluted 1:10 in PBS and centrifuged at 500 × *g* for 5 min. Samples were resuspended in 300 µL PBS, refrigerated, and analyzed within 2 h of protocol completion. A total of 1 × 10^5^ events were recorded for each sample. Instrumentation controls remained fixed for all measurements. Following compensation and unmixing, a P1 gate was applied to isolate the normal population of the upper bimodal distribution relative to forward- and side-scatter area under the curve (FSC-A, SSC-A) across all samples. A secondary gate (P2) was applied to exclude the non-normal distribution of doublets by maximum forward scatter against area under the curve (FSC—H, FSC-A) across all samples. Quantification of marker expression was facilitated by bisecting plots relative to unstained control signals at a threshold of < 0.5 %. (Supplementary Figure 2).

### 2.10 Teratoma assay and histology

Severe combined immune deficiency (SCID) mice were obtained from Charles River (Wilmington, Massachusetts) and stored in a single-barrier animal facility under approval of procedure by the University of Calgary Animal Care Committee (Faculty of Veterinary Medicine, University of Calgary). All mice were fed ad libitum and maintained on a normal day / night cycle. Mice were anesthetized using veterinary isoflurane. A total of 1 × 10^6^ dissociated hiPSCs in 100 μL of Matrigel (354,277, Corning) diluted 1:2 in 1x PBS and 10 μM Y27632 were injected subcutaneously into the inguinal region on each side of two mice (four injections total) following static serial passage. After 10 weeks, the mice were euthanized and teratomas excised. Excised tissue was fixed in neutral buffered formalin (NBF) for 24 h. The tissue was then processed overnight using an automatic tissue processor and embedded in paraffin wax. The tissue was sectioned using a microtome at 10 μm thickness and stained with H&E. Tissue sections were imaged using a Zeiss AxioScan microscope (AxioScan.Z1).

### 2.11 Immunohistochemistry imaging and processing

Tissue sections were first deparaffinized and rehydrated through a series of ethanol dilutions to distilled water. Antigen retrieval was then performed using a 1-hour incubation in 10 mM sodium citrate solution (pH 6) followed by washes in PBST and PBS before blocking with 5 % BSA for 75 min. Detection of endoderm, mesoderm, and ectoderm tissue was performed using Alexa Fluor 647 anti-GATA4 (#948,606, BioLegend), NL557 anti-Brachyury (#NL2085R, Novis Biologicals), and Alexa Fluor 488 anti-Tubulin β 3 (#801,203, BioLegend) all at 1:50 dilutions. Slides were incubated overnight at 4 °C with the antibodies, washed twice in PBST and once in PBS before mounting and coverslipping using EverBrite Hardset mounting medium containing DAPI (#23,004, Biotium). Tissue sections were imaged with the Zeiss AxioScan.Z1 slide scanner. Fluorescence images were processed in Fiji [23]. Image files were split into individual channels and converted to 8-bit. Median filtering (2 px.) and background removal (50 px. rolling ball) were applied independently to each channel. For primary antibody channels, a lower histogram cutoff was applied at the sum of the mean and standard deviation of negative control images.

### 2.12 Statistics

Three biological replicates were performed for each experiment following optimization, with three technical replicates taken per culture for daily counts and imaging. All reported values are given as mean ± standard deviation. To assess statistical significance, Mann-Whitney U tests were conducted between conditions at two separate error probability thresholds: α = 0.05 (*) and α = 0.01 (**). The symbols * and ** denote significance at the 5 % and 1 % levels, respectively. To compare dynamic to static, samples from all agitation rates were pooled and permutation testing applied to assess significance of randomly selected sample subsets against static.

## 3 Results

### 3.1 Assessment of single-cell inoculation and aggregate preformation protocols

**Fig. 1:**
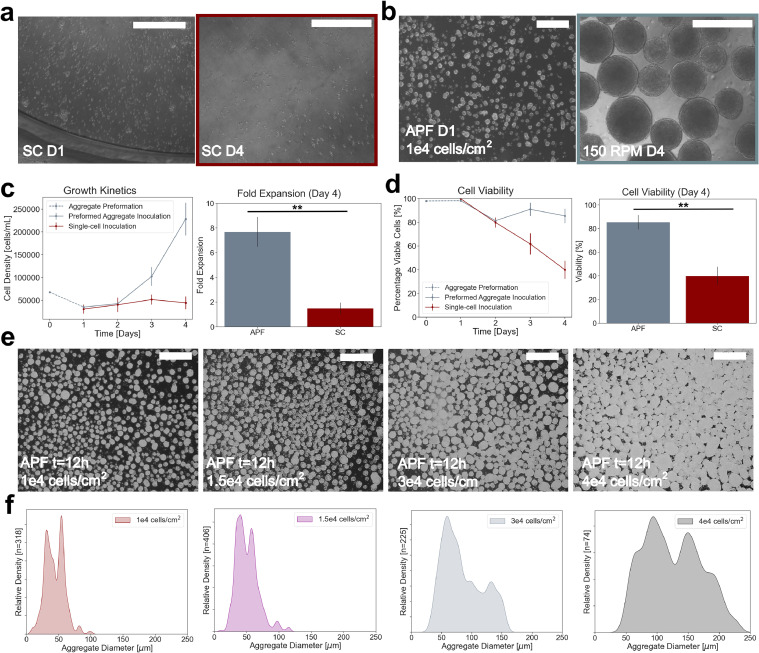
(a) Comparison of scale-down bioreactor outcomes post-inoculation at day 0 and day 4 for single-cell (SC) and aggregate-preformation (APF) conditions. Growth kinetics and viability over culture, alongside day 4 post-harvest measurements (c, d). (e) Morphological comparisons of aggregate preformation in static 12-well plates at *t* = 12 h for cell concentrations ranging from 1 × 10^4^ – 4 × 10^4^ cells/cm^2^. (f) Aggregate diameter distributions following image processing at 12 h following inoculation. All scale-bars are 500 µm. Values are reported as mean ± standard deviation. The symbols * and ** denote error probability thresholds at 0.05 and 0.01, respectively.

Healthy colony formation was observed across static cultures at day 0 (4 days following static inoculation). Following single-cell inoculation and culture in the scale-down reactors, no aggregate formation or significant growth was observed in assessing morphology of samples taken from inoculated vessels (Fig. 1a). Agitation rate was assessed as an influencing factor on aggregate formation *in vitro*. Single cell inoculation did not result in aggregate formation for cells cultured at 60, 80, 100, 120, or 150 RPM (Supplementary Figure 1a). No significant increase in cell density was observed under single-cell inoculated conditions. Preformation of aggregates on plates seeded at 1 × 10^4^ cells/cm^2^ consisted of spherical clusters of cells maintaining high uniformity and acceptable mean aggregate size after 24 h (Fig. 1b). Following dynamic culture of preformed aggregates, morphology showed a well distributed population of larger aggregates, confirming growth and proliferation under scale-down bioreactor conditions. A mean fold expansion of 7.6 was observed for 150 RPM culture following 72 h of agitation after inoculation (Fig. 1c). Cells inoculated following aggregate preformation (APF) retained viability at or above 80 % over the course of batch culture at 150 RPM (Fig. 1d). Daily cell density measurements of single-cell inoculated culture corroborated morphological findings, with cell viability declining to a final reported value of 40 %. Propagation of dynamic culture to day 5 (96 h following preformation) resulted in mean fold expansion of 15.8 and mean viability of 79.9 % (Supplementary Figure 1c).

Given the observed criticality of aggregate preformation in enabling growth and proliferation of hiPSCs in scale-down culture, characteristics of self-aggregation were studied by inoculating incremental cell densities in well plates. Lower inoculation densities resulted in a larger number of small diameter preformed aggregates, whereas larger inoculation densities resulted in formation of a smaller number of large aggregates (Fig. 1e). For inoculation densities of 1 × 10^4^ and 1.5 × 10^4^ cells/cm^2^, tightly controlled distributions of aggregates with slight bimodal tendency around a mean diameter of 46 µm and 53 µm were respectively observed. Further increasing cell density resulted in higher mean aggregate diameter, and larger variance around the mean. A mean diameter of 93 µm was observed at a cell density of 3 × 10^4^ cells/cm^2^. At a cell density of 4 × 10^4^ cells/cm^2^, aggregates were distributed around a mean of 132 µm (Fig. 1f). Total variance in aggregate diameter increased with inoculation density, with reported values of 105 µm, 123 µm, 146 µm, and 229 µm, respective of the inoculation densities assessed. All aggregate preformation was assessed at 12 h. Importantly, no significant change in size distribution was observed following 24 h, though observed number of aggregates was lower.

### 3.2 Optimization of hiPSC proliferation as preformed aggregates in dynamic culture

**Fig. 2:**
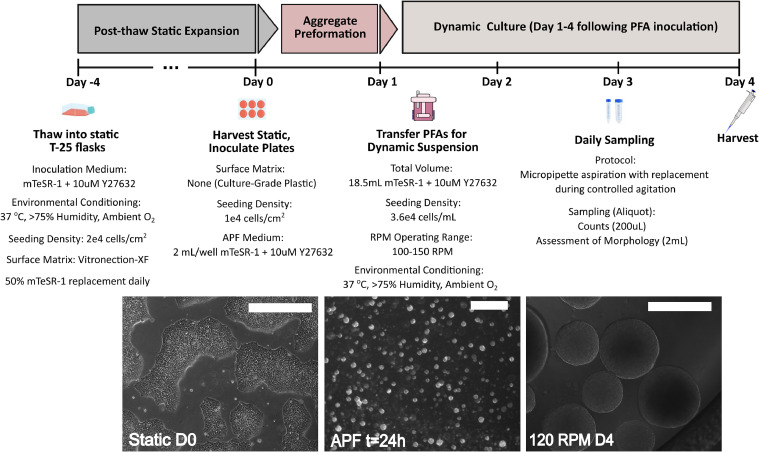
(D-4 to D1) Pre-expansion protocol, including preparation of preformed aggregates for dynamic inoculation. (D1-D4) Dynamic expansion protocol, including manual sampling strategy for assessment of growth kinetics and morphology over culture time course. All scale-bars are 500 µm.

**Fig. 3:**
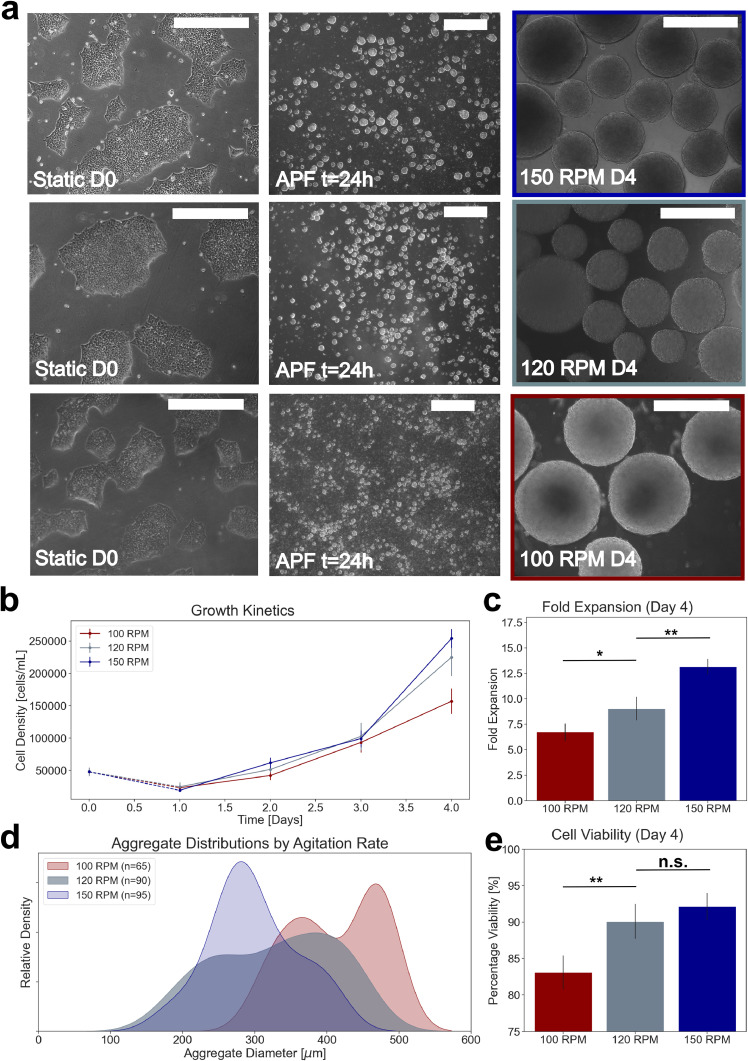
(a) Characteristic morphology of static colonies prior to aggregate formation, preformed aggregates inoculated for each agitation rate, and resulting aggregates on day 4. (b) Growth kinetics observed during expansion, coupled with calculated fold expansion. (c) Fold expansion by agitation rate. (d) Aggregate size distributions derived from image-processing by agitation rate on day 4. (e) Cell viability by agitation rate on day 4. All scale-bars are 500 µm. Values are reported as mean ± standard deviation. The symbols * and ** denote error probability thresholds at 0.05 and 0.01, respectively.

Optimized protocols used to study the effects of agitation rate are shown in Fig. 2. Morphological assessment confirmed that mean aggregate size at day 4 was inversely proportional to the agitation rate used (Fig. 3a). Growth kinetics suggest similar growth from days 1–3 across agitation rates, followed by significantly improved growth at 150 RPM by day 4 (Fig. 3b). Mean fold expansion was lowest in 100 RPM cultures (6.8 ± 0.75), and highest (13.2 ± 0.81) in 150 RPM cultures (Fig. 3c). At 120 RPM, the distribution of aggregates by diameter throughout culture was broadest, suggesting the highest variance in morphological characteristics (Fig. 3d). Mean aggregate diameters and standard deviation of 425 ± 87 µm, 312 ± 113 µm, and 283 ± 69 µm were observed for 100, 120, and 150 RPM cultures following expansion, respectively. Cell viability was greater than 80 % for all cultures following single passage expansion of cells as preformed aggregates, with the highest mean viability observed in 150 RPM cultures (Fig. 3e).

### 3.3 Validation of characteristic and functional pluripotency

**Fig. 4:**
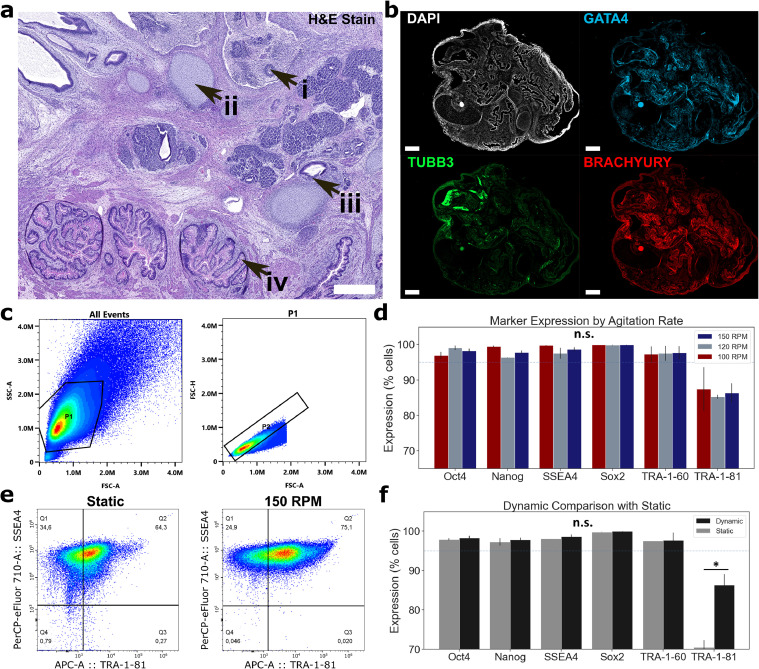
(a) Histological section of excised teratoma. Regions of interest are highlighted: (i) early neural rosette-like formation, (ii) chondrocyte-like cells, (iii) organization resembling glandular epithelium, (iv) organization of intestinal Paneth- and goblet-like tissue. Scale bar is 500 µm. (b) Fluorescence images for lineage differentiation markers of endoderm (GATA4), mesoderm (Brachyury), and ectoderm (TUBB3) in teratoma. All scale bars are 500 µm. (c) Visualization of P1 and P2 gating relative to a single sample. (d) Comparison of marker expression by agitation rate. (e) Comparison of heterogeneity and relative expression of TRA-1–81 against SSEA-4. (f) Comparison of marker expression between pooled dynamic and static single-passage culture. The symbols * and ** denote error probability thresholds at 0.05 and 0.01, respectively.

Teratoma formation was confirmed across all injection sites. Histological examination of H&E-stained sections confirmed the presence of all three primary germ layers (Fig. 4a). Observations included early neural rosette-like formation (i), chondrocyte-like cells (ii), tissue resembling glandular epithelium (iii), and intestinal-like structures suggestive of Goblet- and Paneth-like tissue (iv). Fluorescence images corroborated histological results with positive expression of GATA4, TUBB3, and Brachyury observed (Fig. 4b). Flow cytometry analysis confirmed core pluripotency across populations studied. P1 and P2 gates applied across samples are shown in Fig. 4c. Oct4, Sox2, Nanog and SSEA4 were maintained at >95.9 % expression for static and all agitation rates across biological replicates (Fig. 4d). No significant differences in marker expression were observed between agitation rates. Dynamic culture showed both a shift in distribution towards positive expression and increased homogeneity relative to static when assessed against SSEA-4 (Fig. 4e). TRA-1–81 increased significantly in dynamic conditions relative to static. Serial passaging of cells following dynamic harvest to static conditions confirmed that no significant difference in morphological characteristics, marker expression, or doubling time were observed (Supplementary Figure 1d-f).

## 4 Discussion

### 4.1 Aggregate preformation critically facilitates proliferation in the scale-down environment

It is clear from the results that this scale-down environment, despite being sufficient for the maintenance and proliferation of aggregates in culture, does not support the formation of aggregates from single cells in suspension. This drawback is overcome by effectively facilitating the self-organization of aggregates in static well plates prior to dynamic suspension. Highlighting self-organization in the context of aggregate preformation is pertinent for a multitude of reasons. Early experiments done by our group had utilized non-adherent well plates to facilitate aggregate formation without the risk of being unable to rescue aggregates that had adhered to the culture surface (Supplementary Figure 1b). These aggregates organized poorly, had considerably worse size distributions and aggregate uniformity, and no optimal conditions were observed relative to inoculation density. Interestingly, the inclusion of an adherent surface without viable growth substrate improved aggregate formation remarkably.

Observation of self-organization demonstrates some measure of migratory behavior from the cells. Uniformly distributed single cells organize into aggregate clusters with their closest neighbours (Fig. 1e). This suggests some localized intercellular signaling and migratory behavior is present. It is important that these behaviors be considered in the context of medium composition. Y27632 plays a fundamental role in maintaining a viable cell population prior to organization into embryoid bodies [24]. Interestingly, while preventing dissociation-induced apoptosis in the population, rho kinase (ROCK) inhibition also modulates cytoskeletal regulation, migration, and colony formation [25]. These effects have been shown to modify both self renewal capacity and differentiation potential [26,27]. In our work, aggregate preformation and dynamic culture media were supplemented with ROCK inhibitor, drawing a parallel with protocols we have developed for larger scale systems under single-cell inoculation conditions [15,19]. While aggregate preformation outcomes were deemed sufficient for this study, we believe further work is warranted to better understand the interplay between ROCK-induced cell survival pathways and alteration to intercellular organizational networks, given the existing evidence in literature and the importance of these suspended structures in facilitating hiPSC proliferation (and cell fate trajectory) *in vitro* [[24], [25], [26], [27]].

### 4.2 Aggregate preformation reduces lag phase and influences aggregate dynamics in conjunction with agitation rate

With regards to growth and proliferation of the cell population following preformed aggregate inoculation, there is no notable lag phase despite some cell loss following dynamic suspension on day 1 across agitation rates (Figs. 2c-d, 4b). This suggests that perhaps the lag phase observed in larger scale single-cell inoculation conditions is largely associated with cell collision, clustering of cells into their respective aggregates, and adaptations that are being made by the cells to facilitate a localized niche to facilitate maintenance and proliferation under the hydrodynamic conditions present in the system. It is also important to consider the hydrodynamic force contribution to growth and proliferation, as the lack of lag phase is not observed in larger scale systems under similar study conditions [28]. Viability was comparable across agitation rates on day 4, and the highest fold expansion was observed at 150 RPM. This was coupled with a normal distribution of smaller aggregate sizes. It is expected that aggregate preformation parameters can be used to modulate distribution characteristics over the course of the bioprocess, though this should be tested by further work to elucidate the effects of gradient dynamics and density characteristics on the localized aggregate niche, and the contributions from system setpoints. Given the intentions of this system to be utilized in elucidating short temporal phenotypic dynamics for our current studies, fed-batch culture protocols were not implemented across agitation rates. Extending expansion time within-passage is entirely feasible, though previous work confirms that a fed-batch approach is necessary to facilitate healthy phenotype during continued propagation [15,19].

Another important variable that must be considered with respect to extended propagation time is aggregate size distributions – given that aggregates do not form from single-cell interactions in this culture system, resulting aggregate diameters are higher over the period of culture used in this study relative to single-cell inoculated processes [14,18,19]. Interestingly, a recent study assessing marker expression of downstream pancreatic endocrine cells derived from hiPSCs found comparable results from static preformed embryoid bodies when compared to those expanded from single-cells in stirred suspension bioreactors [29]. This suggests that preformation does not significantly alter marker expression post-differentiation when applied during cell source material expansion. To what extent phenotype and function may diverge between such conditions is a question requiring a greater degree of characterization beyond conventional approaches to cell identity.

### 4.3 Cultured hiPSCs maintain pluripotency under scale-down conditions and following dynamic-to-static serial passage

Flow cytometry results provided evidence to support maintenance of pluripotency markers Oct4, Sox2, Nanog, and SSEA4, alongside enhanced expression of TRA-1–81 following a single passage in dynamic culture for all agitation rates relative to static. Teratoma assay results further confirmed functional pluripotency by trilineage differentiation. While continuous serial passaging in the bioreactors was not explored herein, a single serial passage to static was performed to assess acutely adapted growth kinetics and morphology. Serial passaging in static following culture under dynamic conditions reliably returned TRA-1–81 expression near to pre-expansion static levels, suggesting modulation of TRA-1–81 is induced by either the chemically defined static culture substrate or agitation. The results were not significantly different than those observed in static culture prior to dynamic expansion. There is plenty of room to explore serial passaging effects on hiPSC integrity and trajectory in combination with hydrodynamic characteristics of agitation relative to reactor geometry. This work needs to be coupled thoughtfully with considerations around alterations to phenotype resulting from the myriad variables that are applied in culture both within and beyond the vessel characteristics. These include influences on gene regulatory networks, epigenetic landscape, metabolism, and extend to donor and reprogramming characteristics of the cell source material [30].

### 4.4 Limitations of this study and future work

While the authors are confident that this work presents a significant step towards our goals of understanding the cause and effect of the artificial niche environment on hiPSC cell state dynamics, there are a number of limitations to address herein. Importantly, this study was conducted as a single-passage assessment. While serial passaging to static was carried out, the results of our study are not indicative of the potential for aberrant outcomes in the cell population. Future work needs to be mindful of the potential for culture adaptation and subsequent alteration to cell state networks, that may give rise to single nucleotide variations or epigenomic aberrations [31,32].

Further, our study does not include computational fluid dynamics (CFD) simulations or other approaches to quantitatively assess the hydrodynamics of the environment beyond characterizing broad functional outcomes in the hiPSC population. These studies, alongside validation at scale are undoubtedly integral to effective scale-up and would provide additional evidence under which to study the effects on hiPSC cell state *in vitro* as a function of the artificial niche [33,34]. It must also be highlighted that the system used in this study is one of many configurations; Unbiased assessment of dynamic culture modalities to quantitatively assess optimal conditions for growth and phenotype in the process environment needs to be considered given the importance of system design on the environmental parameters governing control over the cell population.

## 5 Conclusions

We have reported herein a strategy for the successful scale-down implementation of an hiPSC expansion bioprocess for the purpose of parallelizing the process environment to enable efficient high-throughput expansion bioprocessing. Our results present an optimized protocol to facilitate efficient and parallelizable studies at research scale, using an approach conducive to scale-up. We have reported aggregate preformation and cultivation distributions, and validated pluripotent phenotype by marker expression and teratoma formation. These results will allow us and like-minded researchers to better elucidate phenotype and validate hiPSC cultivation approaches at a scale advantageous for rapid research, development, and optimization. The goal of this research is to accelerate discovery for successful clinical translation in regenerative medicine with a foundation for efficient manufacturing scale-up. Furthermore, we have presented perspective on interactions within the bioprocessing niche that we believe are critical to the improvement of hiPSC cultivation strategies in the quest to manufacture highly effective therapeutics for regenerative medicine. Further work by our group will utilize our developed approach to examine the impact of artificial niche dynamics on hiPSC cell state, and their implications for phenotype and function in therapeutic derivatives.

## Funding sources

This funding was supported by the Canadian Institutes of Health Research (CIHR) [PG/201909], Natural Science and Engineering Research Council of Canada (NSERC) [RGPIN/418976].

## CRediT authorship contribution statement

**James Colter:** Writing – review & editing, Writing – original draft, Visualization, Project administration, Methodology, Investigation, Formal analysis, Data curation, Conceptualization. **Tiffany Dang:** Writing – review & editing, Validation, Resources, Methodology. **Julia Malinovska:** Writing – review & editing, Validation, Resources, Methodology. **Jessica May Corpuz:** Writing – review & editing, Resources, Methodology, Investigation, Formal analysis. **Dora Modrcin:** Resources, Investigation, Formal analysis. **Roman Krawetz:** Supervision, Project administration, Funding acquisition, Conceptualization. **Kartikeya Murari:** Supervision, Project administration, Funding acquisition. **Michael Scott Kallos:** Supervision, Project administration, Funding acquisition.

## Declaration of competing interest

The authors of this publication collectively declare that the work described herein has not been published previously, nor is this article under consideration for publication elsewhere. The article's publication is approved by all authors and by the responsible authorities at the University of Calgary. If accepted, this article will not be published elsewhere in the same form, in English or in any other language, including electronically, without the written consent of the copyright-holder. The authors declare that we have no competing interests, and no artificial intelligence tools were used to carry out any aspect of this work.

## Data Availability

All material available upon request to the lead principal investigator (mskallos@ucalgary.ca) or corresponding author (jdcolter@ucalgary.ca).

## Appendix Supplementary materials

Image, application 1
