# Supplementary material for: Scale-down optimization of a robust, parallelizable human induced pluripotent stem cell bioprocess for high-throughput research
Source: Biotechnol Rep (Amst). 2025 May 22;47:e00900. doi: 10.1016/j.btre.2025.e00900 (PMC12164017; doi:10.1016/j.btre.2025.e00900)
Supplement: Supplementary file 1 [file mmc1.docx]

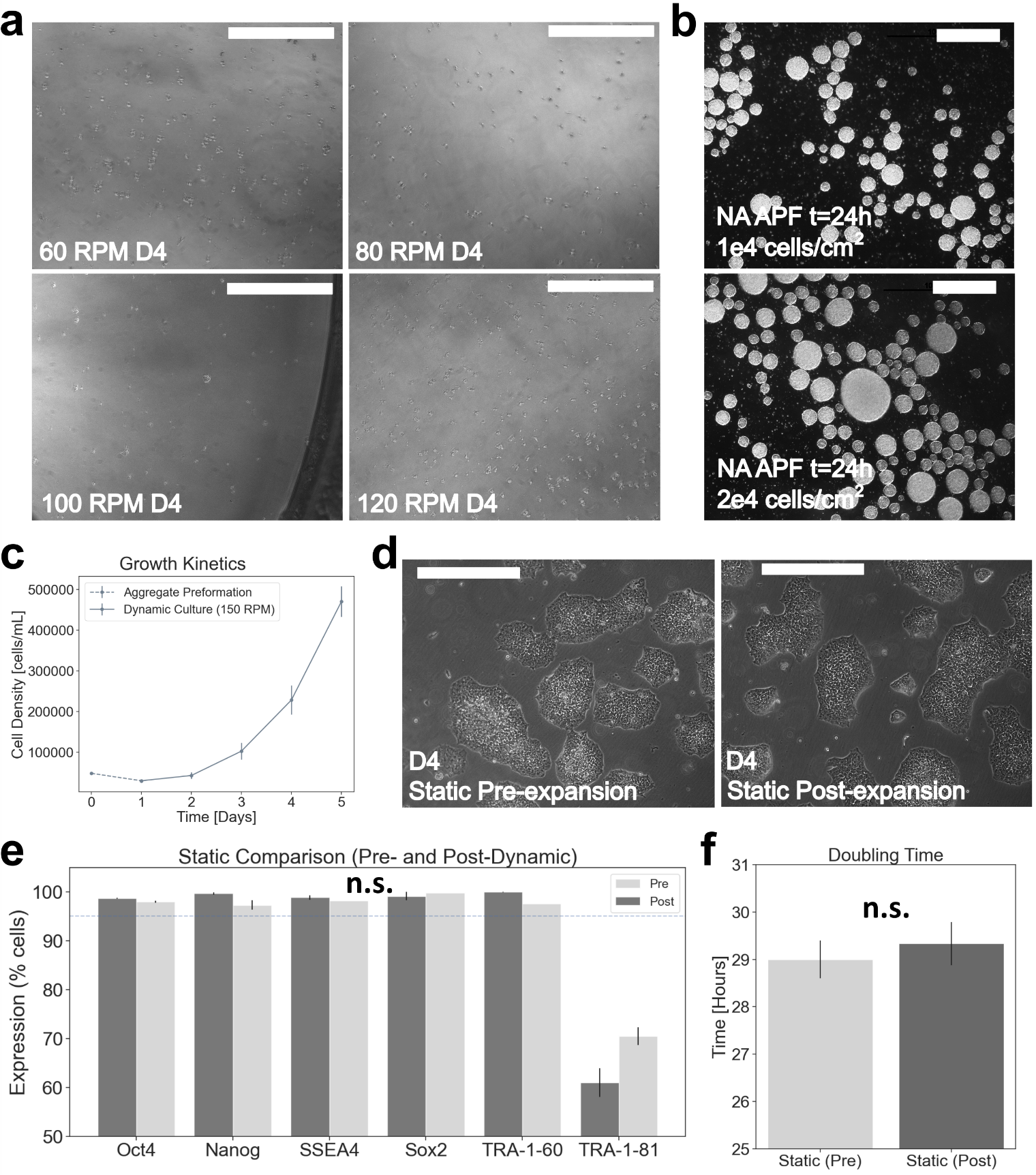


Supplementary Figure 1. (a) Day 4 morphology for dynamic single-cell inoculated cultures at 60, 80, 100, and 120 RPM. (b) Aggregate morphology following organization in non-adherent well plates. (c) Growth kinetics for dynamic culture extended to day 5. (d) Morphological characteristic comparison following post-dynamic culture of cells in static. (e) Marker expression for pre- and post-dynamic expansion in static. (g) Doubling time for single passage pre- and post-dynamic static culture.


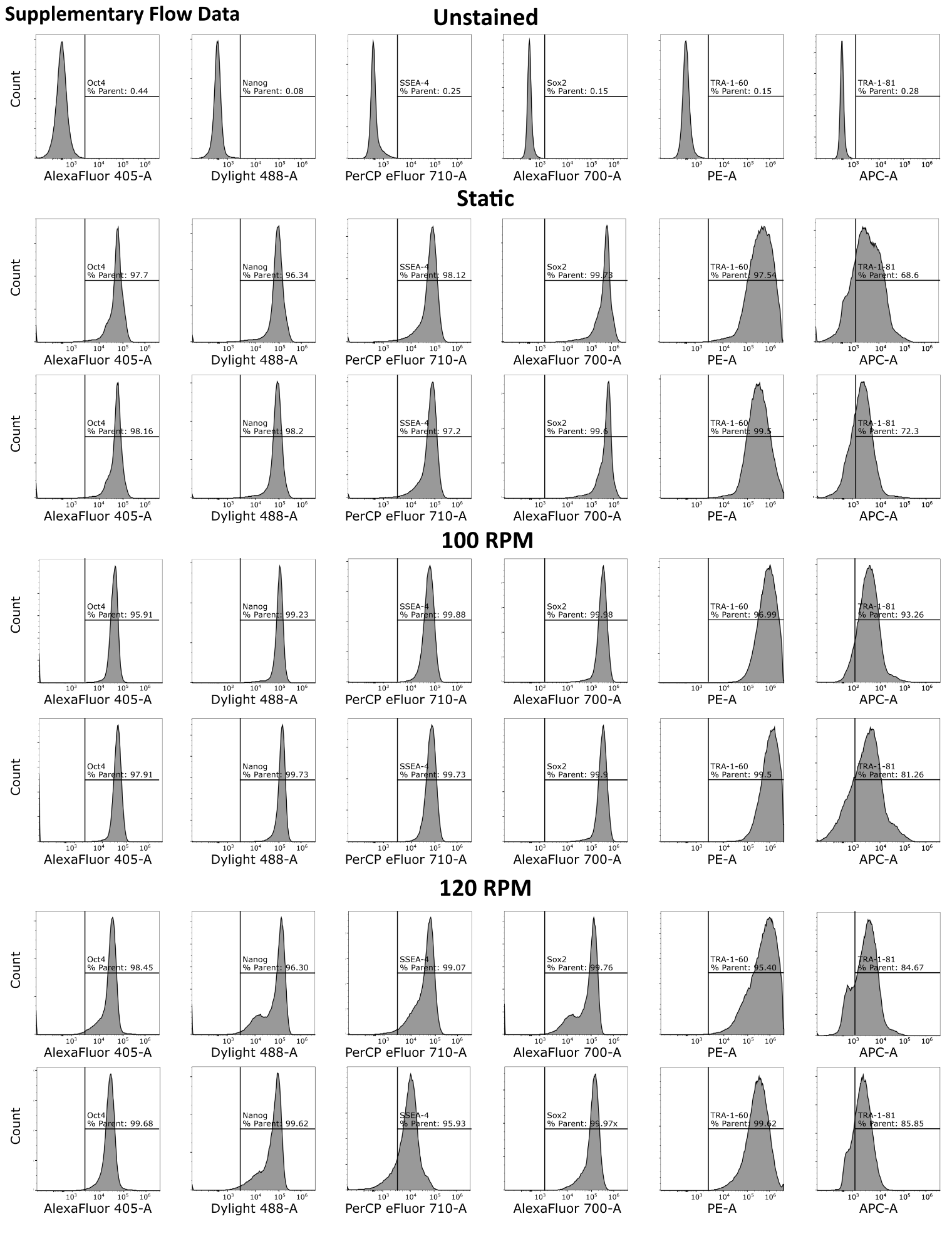


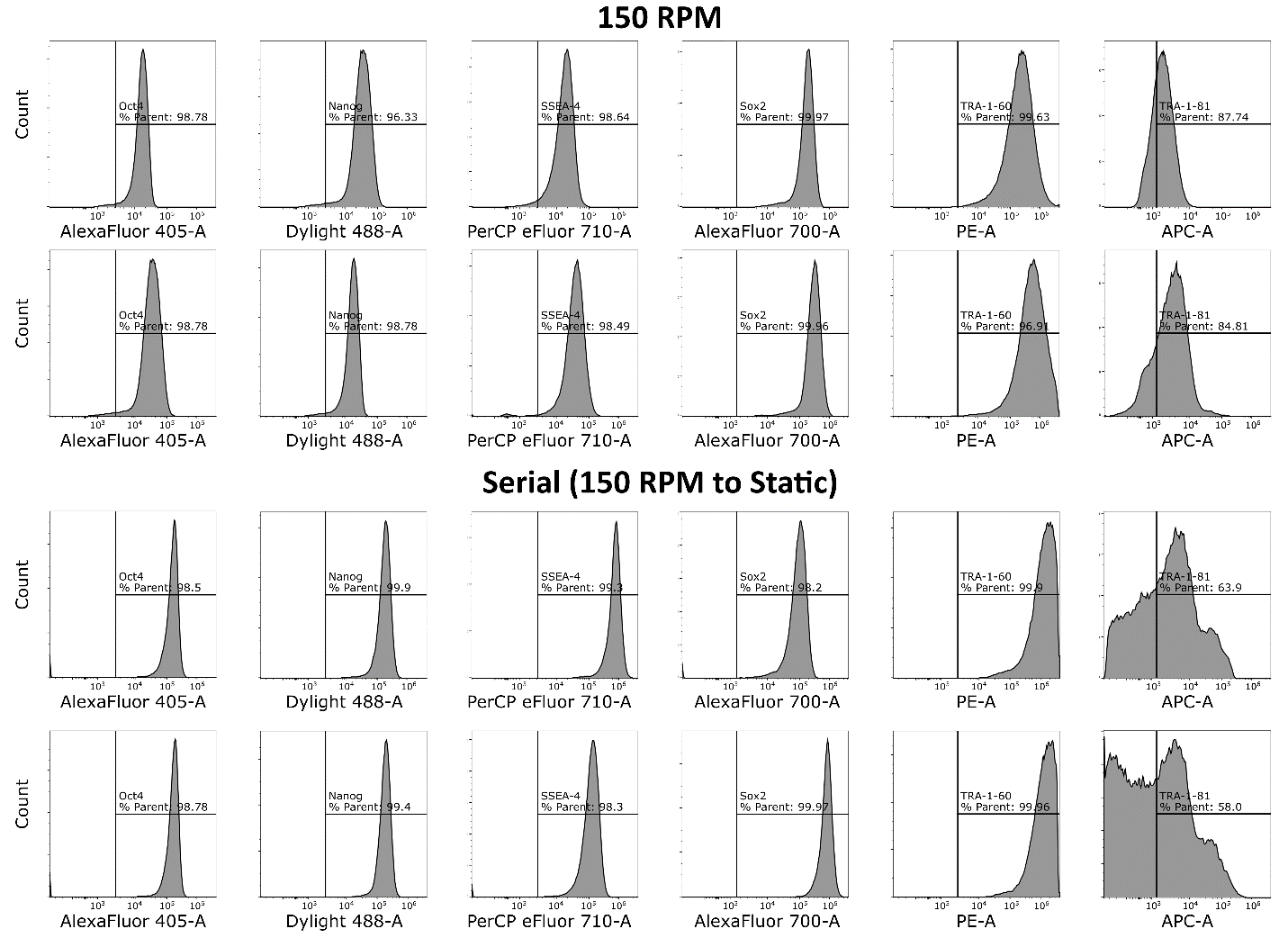


Supplementary Figure 2. All flow cytometry data obtained for expression of Oct4, Nanog, SSEA-4, Sox2, TRA-1-60, and TRA-1-81 by condition and passage. Positive cutoffs are shown relative to unstained controls (top of figure).


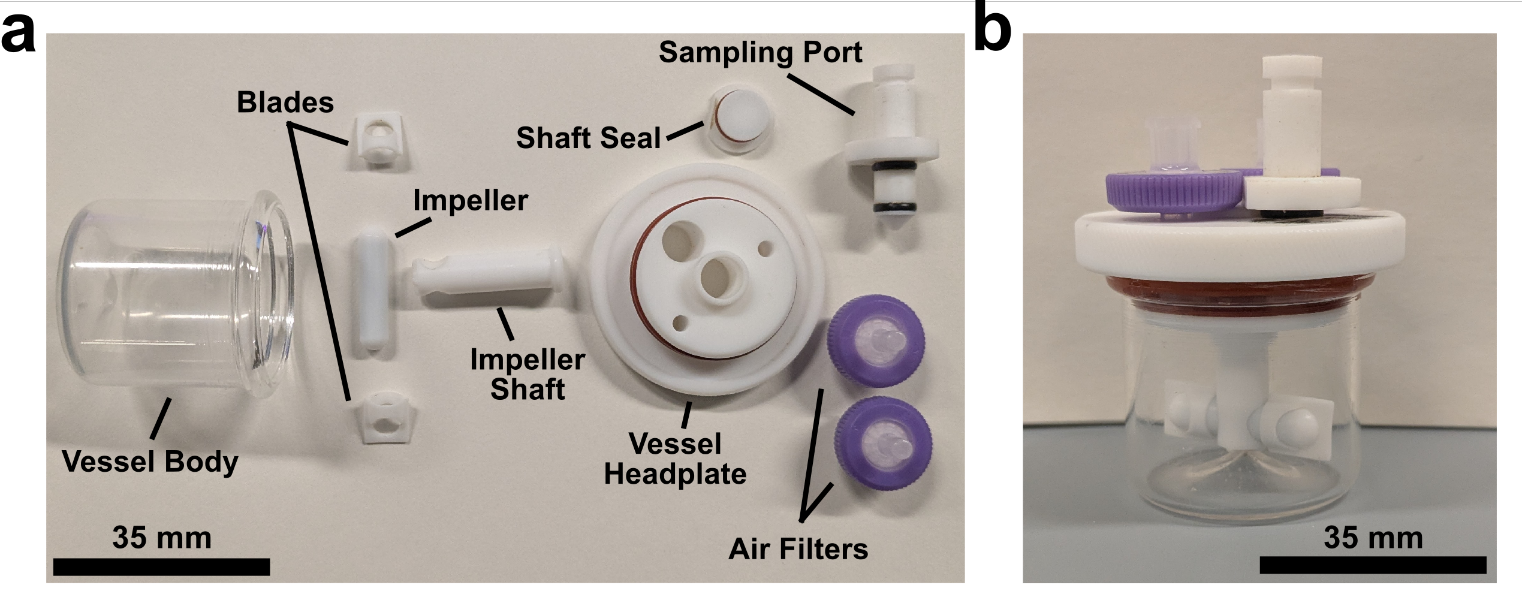


Supplementary Figure 3. (a) Individual components of the scale-down reactor. (b) Assembled reactor.
